# The Psychical Element in Some Functional Disturbances and Its Bearing upon Treatment

**Published:** 1909-01-16

**Authors:** William P. S. Branson

**Affiliations:** Assistant Physician, Royal Free Hospital


### Hospital Clinics.

### THE PSYCHICAL ELEMENT IN SOME FUNCTIONAL DISTURBANCES AND ITS BEARING UPON TREATMENT.

By WILLIAM P. S. BRANSON, M.D., M.R.C.P., Assistant Physician, Royal Free Hospital.

It is a commonplace that the clinical picture of any given disease varies with each victim of it, and physicians in all ages have continued to preach the virtue of studying the individual patient rather than the disease under which he labours. In this respect diseases resemble those composite photographs in which at the humour of the photographer the same trunk and limbs are made to support a variety of headpieces: for the trunk and limbs of an organic malady are represented by the actual dislocation of function produced by the lesions, while the clinical representation or face is never twice alike, since this is dictated by the mental and moral endowments of the individual; that is to say, by his personality. Take, for example, lobar pneumonia, a well-defined disease, whose trunk and limbs, according to our figure, are substantially the same always. They are, in brief, dyspnœa, and a bacterial intoxication evidenced, and in some degree to be measured, by fever. Yet how differently do people sustain attacks of pneumonia!

These considerations apply to all organic diseases. We cannot accept the symptoms of an invalid as the natural expression of a given lesion, for we know how much the clinical representation of diseases is varied by differences in the mental make-up of those who suffer from them. As a rule the graver the physical disability produced by any lesion the less prominent are the mental components of the clinical picture; for the activity of the mind seems to be inhibited in the presence of imminent physical perils, such, for example, as acute general peritonitis. On the other hand, the less intrinsically grave the disorder the more pronounced become the mental components of the disease-picture until, commonly enough, a trifling physical basis of complaint is completely overlaid and obliterated by symptoms of ideogenic origin, by auto-suggestions and phantoms of the mind. The reader will have no difficulty in supplying from his daily experience examples of this sorry faculty to which men and women are continually mortgaging their happiness; but I may cite as a common type the man who, in the jargon of the time, is "full of uric acid." Look into the history of the man, and it is likely that you will find something of this sort. Ten, perhaps twenty, years ago, being troubled with some vague articular stiffness, or a conjunctivitis, or eczema, he consulted a doctor, and learned that he was the subject of "irregular gout." Being an intelligent person and always rather unduly concerned with his internal mechanism he has made a study of the literature of uric acid, and has been repaid by the conviction that he is the victim of an inborn error of metabolism. Thenceforth he is a valetudinarian, constantly on the alert for symptoms. Each passing ache or other anomaly of sensation, each infinitesimal oscillation in the buoyancy of his spirits, all such things are now gloomily noted and deplored as manifestations of the malign compound at work within him; and the chief interest of his life centres round the expulsion of his putative enemy by rigidity in diet, by baths, by drugs, and the whole armament of therapeutics. The insignificant disorder which originated the disastrous sequence has long since disappeared and been forgotten: its memory has been trodden out of sight by the host of auto-suggestions to which it, and the careless remark of the physician, have given birth and liberty.

The mental element in the clinical pictures of organic disease becomes, as I have said, more prominent as the physical disability becomes less; it is not surprising, therefore, to find that the mental component reaches its maximum in that class of disease in which an organic basis for the symptoms either does not exist or is so slight as to elude detection. I mean the class of "functional disturbances." In cases of this class the mental element is admittedly a paramount consideration, and any treatment inaugurated without due regard for this circumstance must be faulty in principle. My present concern is to show that a good many common symptoms properly to be considered functional disturbances pass undetected in these material days, and, receiving from the medical man the stamp of reality, are confirmed and rendered enduring by the very treatment which is aimed at the dissipation of them.

The commonly received definition asserts that a functional disturbance is an anomaly of function not to be explained on the score of structural alteration in the parts concerned, and it is well known that the greatest sufferers from functional disturbances are those of a neurotic temperament. The keynote of the neurotic temperament is an anomalous, but generally exaggerated, responsiveness to stimuli of all kinds, manifesting itself not only in the lower reflex functions which lie under the control of spinal centres like that for vaso-motor regulation, but also in the higher functions of the brain—for instance, those concerned with the perception of sensations. It follows, therefore, that the neurotic person may be expected to react to stimuli whose intensity is too slight to produce any response at all in a normal individual, a circumstance offering a possible explanation of some of the random and apparently causeless manifestations of functional disturbance. Let me illustrate my meaning by an example. The act of defæcation is normally carried out in response to a stimulus originated by an accumulation of fæces in the rectum. When this accumulation reaches a certain degree of bulk an impulse is transmitted to the lumbar centre controlling the process of defæcation, and the result is the muscular activity of the rectum, which leads to an evacuation. But it is no rarity to meet with a condition sometimes called the "neurotic rectum." Here the lumbar centre seems to be in a state of unstable equilibrium; its sensitiveness to stimulation varies from day to day. In consequence, on one day a succession of imperious impulses and frequent evacuations will result from small collections of fæces insufficient to excite any impulse at all in a normal individual; while on another day a phase of lethargy on the part of the centre results in constipation, both the diarrhœa and the constipation being "functional disturbances."

So much for functional disturbances based upon an anomalous response of the organism to physical stimuli. I pass now to observe that somatic functions, even those which, like defæcation, are commonly carried out in response to a physical stimulus, can be thrown into operation by stimuli of psychical origin. Note, for example, the laxity of the bowels produced by certain kinds of excitement, particularly the anxious expectation of an ordeal such as a race, or any public appearance at which personal reputation is at stake. Note the sense of nausea, which may actually proceed to vomiting, occasionally produced in imaginative people by the spectacle of something revolting, or even by the perusal of a revolting description. Moreover, an emotional stimulus can not only throw a normal function into operation; it can on occasion modify or actually inhibit it. For instance, the normal response to a distended bladder or a loaded rectum is frequently inhibited by over-solicitude directed towards the due discharge of the function. Every medical man knows how often patients called upon to pass urine for examination fail in doing it so long as they remain under the doctor's eye, and this even though the bladder be full; and how fatal to regularity of the bowels are even trifling emotional states, like the sense of being in a hurry at the time usually devoted to this business. It is quite certain, then, that no view of functional disturbances can be considered satisfactory if it fails to ascribe a large importance to the effect of emotional states upon bodily functions.

The next point I wish to make seems to me of the utmost moment in connection with the treatment of that large class of functional disturbances which finds clinical expression in vague internal sensations, especially those originating in the abdomen. It is that all sensations are best appreciated when the attention is strained expressly for their appreciation. Conversely, when attention is profoundly distracted, sensory stimuli of considerable, and even great, intensity may fail to affect consciousness. For instance, the same man may have a sense of touch delicate enough to appreciate the lightest stroking with a hair while he is on the look out for it, and yet receive a serious wound in a scuffle or exciting game without knowing it until his mental distraction has passed away. The importance of this consideration lies in the fact that although in the ideal state of health the vital functions of the body find no expression in the consciousness of the individual, yet in a certain type—namely, the neurotic—a good many of these vital functions are performed to the accompaniment of sensations, often vague in character, but provocative of considerable discomfort and anxiety. Take, for instance, the middle-aged woman who complains of palpitation, and in whom examination reveals nothing abnormal; commonly enough a little close questioning will show that the palpitation complained of is really nothing more than consciousness of the pulsations of the heart, this consciousness becoming, as one would expect, most tiresome at times when the attention is otherwise idle, as it is in bed, or when the accustomed pulse-rate is disturbed by food or stimulants. No one can fail to be struck by the difference in sensibility towards the vital functions exhibited by different people, and this as regards both the normal and the abnormal performance of them. One man, as I have said, will complain of palpitation if his heart beats a trifle faster than usual, while another may to the examiner manifest a high degree of arrhythmia under circumstances justifying the presumption of organic myocardial changes, and yet be quite unconscious that his heart is beating irregularly. It seems certain that these differences are not so much a matter of variation in the stimuli as in the delicacy of the perceptive faculties at the moment, and it is vital for our purpose to remember that this morbid delicacy of perception can be diminished or enhanced according as attention is or is not distracted. One other matter requires to be mentioned here, though it is almost a commonplace. It is that in some persons, perhaps in most under favourable conditions, anxious expectation of a sensation is capable of originating the sensation apprehended, or at least something which is mistaken for it; either, that is to say, the sensation is purely ideogenic, like those which visit us in dreams, or, though due to an organic cause, it is rendered disproportionate to the stimulus by the apprehension with which it is awaited. For instance, a man who grasps the handles of an electrical machine under the firm conviction that he will receive a shock will often protest that he has received the shock he expected, although it can be proved that no current is passing; the sensation, that is to say, is purely ideogenic: or, on the other hand, the passage of a small current may make him drop the handles, vowing that the shock was severe; that is to say, the enhancement of his perceptive faculty under the influence of apprehension has led him into a misconstruction of the extent of the stimulus to which he has been subjected.

Let me now recapitulate the chief premises which I have attempted to establish.

1. In the clinical representation of every disease there are two elements: (*a*) the disturbance of function, which in its immediate effects is the same, whether it depends upon organic disease or not, and (*b*) the mental superstructure, that is, the effect upon the patient's mind produced not by the disordered function, but by the patient's knowledge that it is disordered, and by his consequent auto-suggestions and fears. It will be clear, I think, that success in treatment turns upon the physician's penetration, and his capacity to determine for any given example which of these elements is the preponderant one.

2. Functional disturbances are the particular heritage of the neurotic, and the characteristic of this class is an enhanced responsiveness to stimuli of all kinds. It is reasonable, therefore, to assume that functional disturbances depend upon the operation of stimuli so trifling that ordinary individuals do not react to them. [The stimulus often enough cannot even be guessed at; but whatever the stimulus, it is a prominent feature of functional disturbances that they are, on the whole, transient.]

3. Most, if not all, of the vital functions can be thrown into abnormal activity, modified or inhibited by emotional stimuli.

4. Attention will enhance the appreciation of sensations; distraction will diminish it. Moreover, anxious apprehension of a sensation is capable of originating, under favourable circumstances, the sensation apprehended.

I will now, for the sake of illustration, briefly apply these considerations to some symptoms derived from the alimentary system, and called chronic indigestion: symptoms which, to my mind, represent for the most part mere disturbances of function, although it is the custom to treat them as organic, and thereby, as I think, in not a few instances to perpetuate what would else have been a passing disorder. The term "indigestion," when used by a patient, may mean lassitude, or sleeplessness, or palpitation, or fifty other symptoms quite remote, as far as one can see, from any disturbance of the digestive system. But it means also certain tolerably well-defined sensations which are related to food and to the alimentary canal —namely, an epigastric uneasiness, sometimes described as pain, sometimes as fulness, or emptiness. It is often associated with a sense of substernal discomfort leading to frequent eructations voluntarily undertaken. We know nothing, unfortunately, about the precise stomachic conditions which determine these sensations. Sometimes the sense of fulness is attended by an increase in the stomach resonance, but quite frequently it is not so, while the sense of emptiness seems to be something quite distinct from that of healthy hunger. We know, I say, nothing about the condition of the stomach in these cases; but the assumed lesion is a "gastric catarrh," that is to say, a real inflammatory change in the mucosa to be subdued by sedatives like bismuth, and by a rigorous dietary. Now our knowledge, as opposed to our assumptions, of organic lesions of the stomach is remarkably small. Simple ulcers and their later mechanical consequences, malignant ulcers, erosions; these can from time to time be demonstrated, but by comparison with the frequency of "chronic indigestion" these lesions are rarities. What, then, of "gastric catarrh"? Arguing by the analogy of mucous membranes more open to investigation than is that of the stomach, one may say that a catarrh depends upon an irritant poison, whether chemical or bacterial; thus we may say with a probability of accuracy that the digestive discomfort following an alcoholic debauch is due to a real gastric catarrh produced by the irritant poison alcohol. But it is an illuminating fact that in cases like these, where the gastric mucosa probably is catarrhal, the complaint is less of epigastric discomfort than of general malaise and anorexia; moreover, the people who suffer most from chronic indigestion are precisely those who are most particular in the avoidance of all irritants. Are we, then, to assume a chronic bacterial infection? We know that in conditions associated with extreme stasis of the stomach-contents micro-organisms appear in the retained material, and it is probable enough that in such circumstances a secondary infection of the gastric mucosa plays some part in the production of symptoms; but there is, so far as I know, no evidence that gastric catarrh is at all a frequent lesion. How, then, is one to account for the frequency of the disturbances in the function of digestion apart from gross dietetic indiscretions. The answer is to be sought, it seems to me, in the considerations to which the earlier part of this paper is devoted. If the stomach is, as I believe, remarkably free from organic lesions, we know that its functions are extremely open to a variety of influences acting by nervous paths. We know, for example, that any painful emotion, whether acute, like horror, or chronic, like the complex emotion we call worry, can inhibit digestion, and that this inhibition is attended by a vague epigastric uneasiness. This being so, it does not place a heavy tax upon imagination to assume that a great variety of trifling causes which escape detection are capable of influencing the functions of the stomach, both motor and secretory, and of producing a consciousness of disordered digestion. Once this point is reached in the evolution of a case of chronic dyspepsia the ideogenic element makes a definite appearance, for the victim, knowing that his "stomach is out of order," is constantly on the watch for indications of its trouble; and this expectation in itself provides him with a pessimistic attitude of mind most inimical to good digestion. Although his symptoms vary from day to day, he grows, on the whole, more and more unhappy, and seeks medical advice. The result is a tonic medicine and a long list of articles of diet which he must rigidly avoid. He avoids them religiously enough, for by this time his thoughts are all day hovering about his epigastrium, while the practical starvation to which he is condemning himself produces a natural constipation, confirming him in his conviction that he is indeed seriously unwell. He next betakes himself in despair to a "stomach specialist," who detects in him a gastric dilatation, and will, according to his professional enthusiasm, merely diet him further, or wash out his stomach, or perform a gastro-enterostomy upon him. This, I submit, is no overdrawn description of many a confirmed case of functional dyspepsia.

But what, it may be said, is to be substituted for the drugging and dieting? The answer is that if a careful analysis of the temperamental constitution of the patient, of his circumstances, and of the symptoms, leads to the conclusion that there is no serious organic disease, the therapeutic indications are to set the patient's mind at rest as far as possible; to urge the wisdom of distracting the attention from the sensations complained of; to give a simple exposition of the effect exercised upon the digestion by worry and such emotions, and to make the dieting and drugging a very secondary matter. If the case is of long standing, and the patient exhausted by his disease (and the treatment he has been giving it), rest and over-feeding must be added, but the first essential is to replace with optimism and healthy stoicism the gloomy outlook which is exhibited by sufferers from chronic dyspepsia, and to distract them from the morbid contemplation of their sensations.

